# Safety, tolerability, and concordance with interferon-γ release assays of a recombinant ESAT6–MPT64 skin test: a phase 1 randomized clinical trial

**DOI:** 10.3389/fimmu.2026.1780294

**Published:** 2026-04-21

**Authors:** Xichao Ou, Guan Liu, Baozhen Peng, Yuehua Li, Hegui Yan, Naihui Chu, Bing Zhao, Yanlin Zhao, Juan Du

**Affiliations:** 1 National Key Laboratory of Intelligent Tracking and Forecasting for Infectious Diseases, National Center for Tuberculosis Control and Prevention, Chinese Center for Disease Control and Prevention (Chinese Academy of Preventive Medicine), Beijing, China; 2 Phase I Clincal Trial Center, Wuhan Pulmonary Hospital(Wuhan Institute for tuberculosis control), Wuhan, China; 3 Tuberculosis Department, Beijing Chest Hospital, Capital Medical University, Beijing, China

**Keywords:** diagnostic accuracy, ESAT6-MPT64, IGRA concordance, MPT64, mycobacterium tuberculosis, phase 1 clinical trial, recombinant skin test

## Abstract

**Background:**

Accurate and cost-effective screening for Mycobacterium tuberculosis (Mtb) infection remains a global challenge. While Interferon-γ Release Assays (IGRAs) offer high specificity, their widespread deployment is hindered by high costs and technical complexity. Conversely, the traditional Tuberculin Skin Test (TST) lacks specificity due to BCG cross-reactivity. Emerging recombinant skin tests predominantly rely on the ESAT6-CFP10 antigen combination. To potentially broaden the antigenic repertoire and enhance diagnostic sensitivity, we developed a novel recombinant fusion protein incorporating MPT64 (from Region of Difference 2, RD2) alongside ESAT-6. This study represents the first-in-human evaluation of the ESAT6-MPT64 (EM) skin test.

**Methods:**

This single-center, randomized, open-label, dose-escalation Phase 1 clinical trial (Registration: ChiCTR2500112887) enrolled 60 participants, comprising 30 healthy controls and 30 patients with active pulmonary tuberculosis (TB). Participants were stratified and randomized into low-, medium-, and high-dose cohorts (n = 10 per cohort/group). The primary endpoint was safety and tolerability. Secondary endpoints included diagnostic performance (sensitivity and specificity) and concordance with the T-SPOT.TB assay (IGRA).

**Results:**

The EM skin test demonstrated an excellent safety profile. Adverse events were predominantly mild (Grade 1–2), transient, and self-limiting, with no serious adverse events (SAEs) related to the investigational product reported. In terms of diagnostic performance, the test exhibited robust immunogenicity in active TB patients, achieving a peak sensitivity of 87.0% at 48 hours and maintaining 82.6% at 72 hours post-injection. In healthy controls, the test showed high specificity with a low rate of non-specific reactions (13.3%). Receiver Operating Characteristic (ROC) analysis indicated high diagnostic accuracy, with Area Under the Curve (AUC) values exceeding 0.80 across all dose groups at both 48- and 72-hour reading windows. Furthermore, the EM skin test demonstrated substantial concordance (κ > 0.60) with IGRA results, confirming that the specific DTH response is distinct from BCG vaccination background.

**Conclusion:**

The recombinant EM skin test is safe, well-tolerated, and demonstrates preliminary diagnostic accuracy comparable to IGRAs. By successfully validating the translational utility of the MPT64 (RD2) antigen in a human cohort, this study provides a strong proof-of-concept for the EM skin test as a scalable, specific, and cost-effective immunodiagnostic tool.

## Introduction

Tuberculosis (TB), caused by Mycobacterium tuberculosis (Mtb), persists as a formidable global public health challenge. According to recent estimates, approximately 10.7 million people developed TB in 2024, resulting in an estimated 1.23 million deaths worldwide (1). China, bearing one of the highest TB burdens globally, reported significant incidence and mortality rates in 2024. The targeted screening and preventive treatment of Mtb infection are cornerstones of the World Health Organization’s (WHO) End TB Strategy, essential for interrupting transmission and preventing progression to active disease (2). However, the effectiveness of these interventions is currently limited by the inherent shortcomings of available immunodiagnostic tools, which remain inadequate for accurate risk stratification and mass screening in high-prevalence settings (3, 4).

For over a century, the Tuberculin Skin Test (TST) has served as the primary screening tool. Relying on purified protein derivative (PPD)^-^a heterogeneous mixture of antigens shared among Mtb, Mycobacterium bovis BCG, and various non-tuberculous mycobacteria (NTM)-the TST suffers from poor specificity. This antigenic cross-reactivity frequently results in false-positive reactions in BCG-vaccinated individuals and those exposed to environmental mycobacteria, thereby confounding clinical interpretation and leading to unnecessary therapeutic interventions (5, 6). To address these specificity concerns, Interferon-γ Release Assays (IGRAs), such as QuantiFERON-TB Gold and T-SPOT.TB, were developed. These assays detect T-cell responses to Mtb-specific antigens, primarily Early Secretory Antigenic Target 6 (ESAT-6) and Culture Filtrate Protein 10 (CFP-10), which are encoded within the Region of Difference 1 (RD1) (7, 8). However, despite their improved diagnostic accuracy, IGRAs remain constrained by high cost, technical demands, and the requirement for advanced laboratory infrastructure, limiting their widespread use in resource-limited settings (9–11).

To bridge the diagnostic gap between TST and IGRAs, next-generation skin tests utilizing recombinant Mtb-specific antigens have emerged. These novel assays aim to circumvent the cross-reactivity issues of PPD while retaining the operational simplicity of traditional skin testing. Prominent examples, such as the Diaskintest, SIILTIBCY, and C-TST, typically employ the RD1-encoded antigens ESAT-6 and CFP-10. While Phase 2 and 3 clinical trials have substantiated their diagnostic utility and concordance with IGRAs (12–14), relying solely on the RD1 antigen pair may present limitations. Differences in HLA genotype may cause variations in host immune responses to individual antigens. Therefore, incorporating additional immunodominant antigens to broaden T-cell epitope coverage is a rational strategy for enhancing diagnostic sensitivity (15).

In this context, the MPT64 protein presents a promising candidate. Encoded by the Rv1980c gene within the Region of Difference 2 (RD2), MPT64 is a highly immunogenic secreted antigen restricted to the M. tuberculosis complex and absent in most BCG strains (except for minor subs trains like BCG-Tokyo) and NTMs. Importantly, MPT64 has been shown to induce robust delayed-type hypersensitivity (DTH) responses in Mtb-infected models and to elicit antigen-specific T-cell responses distinct from those induced by ESAT-6 (16). These properties suggest that MPT64 may complement ESAT-6 by expanding antigen recognition and improving the detection of infected individuals. We hypothesized that a novel recombinant fusion protein-integrating the well-characterized RD1 antigen ESAT-6 with the RD2 antigen MPT64 (EM)-could broaden the antigenic repertoire. This combination aims to leverage the dominant epitope targets of ESAT-6 while harnessing the robust immunogenicity of MPT64, thereby potentially enhancing diagnostic sensitivity without compromising specificity.

Preliminary studies in our laboratory have supported this hypothesis, demonstrating that the EM fusion protein elicits specific cell-mediated immune responses in infected animal models (16). Building on these preclinical data, we initiated this Phase 1 randomized clinical trial to provide the first-in-human validation of the recombinant EM skin test. Here, we report the comprehensive assessment of the safety, tolerability, and preliminary diagnostic performance of this novel reagent in both healthy volunteers and patients with active tuberculosis.

## Methods

### Study design

This single-center, randomized, open-label, Phase 1 clinical trial was conducted at Wuhan Pulmonary Hospital (Wuhan, China). The primary objective was to evaluate the safety and tolerability of the novel recombinant ESAT6-MPT64 (EM) skin test, with the assessment of diagnostic performance serving as the secondary objective.

The study protocol was reviewed and approved by the Institutional Review Board (IRB) of Wuhan Pulmonary Hospital. The trial was conducted in strict accordance with the Declaration of Helsinki and Good Clinical Practice (GCP) guidelines. Written informed consent was obtained from all participants prior to enrollment. Independent oversight of the study conduct and safety data was provided by an external Data Safety and Monitoring Board (DSMB).

As this was a first-in-human Phase 1 trial primarily designed to evaluate safety and tolerability, the sample size was determined based on feasibility considerations and the need to ensure adequate safety monitoring, rather than on formal statistical power calculations. In early-phase clinical studies, sample size determination is not typically driven by hypothesis testing, but instead guided by practical, ethical, and safety-related considerations. Accordingly, a total of 60 participants was deemed appropriate to generate sufficient preliminary safety data while minimizing unnecessary exposure of participants.

### Study population and eligibility criteria

The study population comprised two distinct cohorts: patients with active tuberculosis (ATB) and healthy controls (HC). All enrolled participants were aged between 18 and 65 years.

Active Tuberculosis (ATB) Group: Participants were randomly selected from local tuberculosis registers. Inclusion required a microbiologically confirmed diagnosis of pulmonary tuberculosis via culture or nucleic acid amplification testing (NAAT) within the preceding month.

Healthy Control (HC) Group: Eligible participants were healthy adults with no history of TB exposure and no clinical signs or symptoms suggestive of active or latent tuberculosis.

Exclusion Criteria: Key exclusion criteria included: (1) receipt of any live vaccine or EM skin test within the previous 12 months to prevent potential immunological boosting; (2) active disease involving lymphoid organs (unless otherwise specified); (3) current dermatological conditions at the injection site that could interfere with induration measurement; (4) medical conditions posing more than minimal risk during blood sampling; (5) pregnancy, breastfeeding, or unwillingness to use adequate contraception during the trial; and (6) concurrent participation in other clinical trials. (7) known or suspected immunodeficiency, including HIV infection.

### Randomization and blinding

Following the screening of 89 potential candidates, 60 eligible participants (30 ATB patients and 30 HCs) were enrolled in the full analysis set. Participants were stratified by health status and randomized in a 1:1:1 ratio to receive the recombinant EM skin test at one of three dose levels: 2.5 U, 5.0 U, or 10.0 U (per 0.1 mL). The selected dose levels were based on preclinical dose-response studies conducted in Mycobacterium tuberculosis-sensitized guinea pig models, which demonstrated favorable immunogenicity and safety profiles of the EM fusion protein. This allocation resulted in six subgroups, with each dose cohort containing ten ATB patients and ten HCs. The study utilized an open-label design for dose administration.

### Procedures and interventions

Screening Phase (Days -28 to 0): A screening visit was conducted within 28 days prior to randomization to confirm eligibility, obtain informed consent, record demographic and medical history, and perform a comprehensive physical examination and baseline safety blood profiling.

Baseline Visit and Administration (Day 0): Urine pregnancy tests were performed for female participants of childbearing potential. To prevent *in vivo* boosting of immune responses by the skin test, venipuncture for the Interferon-γ Release Assay (IGRA) was strictly performed prior to the administration of the investigational product. The EM antigen was subsequently administered intradermally on the volar aspect of the forearm. Participants were issued diary cards to document local (e.g., pain, pruritus) and systemic adverse reactions.

Follow-up and Assessment: Follow-up visits were scheduled to assess skin test reactions, specifically measuring the transverse diameter of induration and erythema. A final safety assessment was conducted on Day 28 to monitor for delayed adverse events.

### Outcome measures

Safety Assessments: Safety surveillance encompassed both local and systemic adverse events (AEs). AEs were coded according to the Medical Dictionary for Regulatory Activities (MedDRA) using Preferred Terms (PT) and System Organ Class (SOC). For analysis, all systemic AEs observed following administration were conservatively attributed to the intervention. Safety monitoring included comprehensive laboratory assessments (hematology, serum biochemistry, and blood glucose).

### Efficacy endpoints

Skin Test Positivity: Defined as a mean induration diameter of ≥5 mm post-intradermal injection.

Strong Positivity: Characterized by the presence of vesicles, bullae, or necrosis at the injection site, regardless of induration size.

IGRA Concordance: IGRA samples were processed according to the manufacturer’s instructions, with a positivity cutoff of 0.35 IU/mL of interferon-γ.

Diagnostic Performance: Evaluated by comparing detection rates between the bacteriologically confirmed ATB group and the HC group, and by assessing concordance with IGRA results.

### Statistical analysis

Continuous variables were summarized using means ± standard deviations (SD) or medians with interquartile ranges (IQR), while categorical variables were expressed as frequencies and percentages.

Diagnostic Accuracy: The ability of the EM skin test to distinguish ATB patients from HCs was evaluated using Receiver Operating Characteristic (ROC) curve analysis. The Area Under the Curve (AUC) was calculated for each dose cohort, with the optimal diagnostic cutoff determined by maximizing the Youden index. Test positivity rates were reported with 95% Confidence Intervals (CIs).

Concordance Analysis: Agreement between the EM skin test and IGRA was assessed using Cohen’s kappa (κ) coefficient. Kappa values were interpreted as: poor (κ ≤ 0.40), moderate (0.40 < κ ≤ 0.60), and substantial/good (0.60 < κ ≤ 1.00).

Analysis Sets: The safety and primary efficacy analyses included all randomized participants who received at least one dose of the investigational product (Safety Set).

All statistical tests were two-sided, with a P-value of <0.05 considered statistically significant. Analyses were performed using SAS software (version 9.3, SAS Institute, Cary, NC, USA), and visualizations were generated using GraphPad Prism (version 6.4, GraphPad Software, San Diego, CA, USA).

### Role of the funding source

The funder participated in the study design, data analysis, interpretation, and manuscript preparation but had no role in data collection. The corresponding author had full access to all data and held final responsibility for the decision to submit for publication.

## Results

### Demographic characteristics

**Figure 1:**
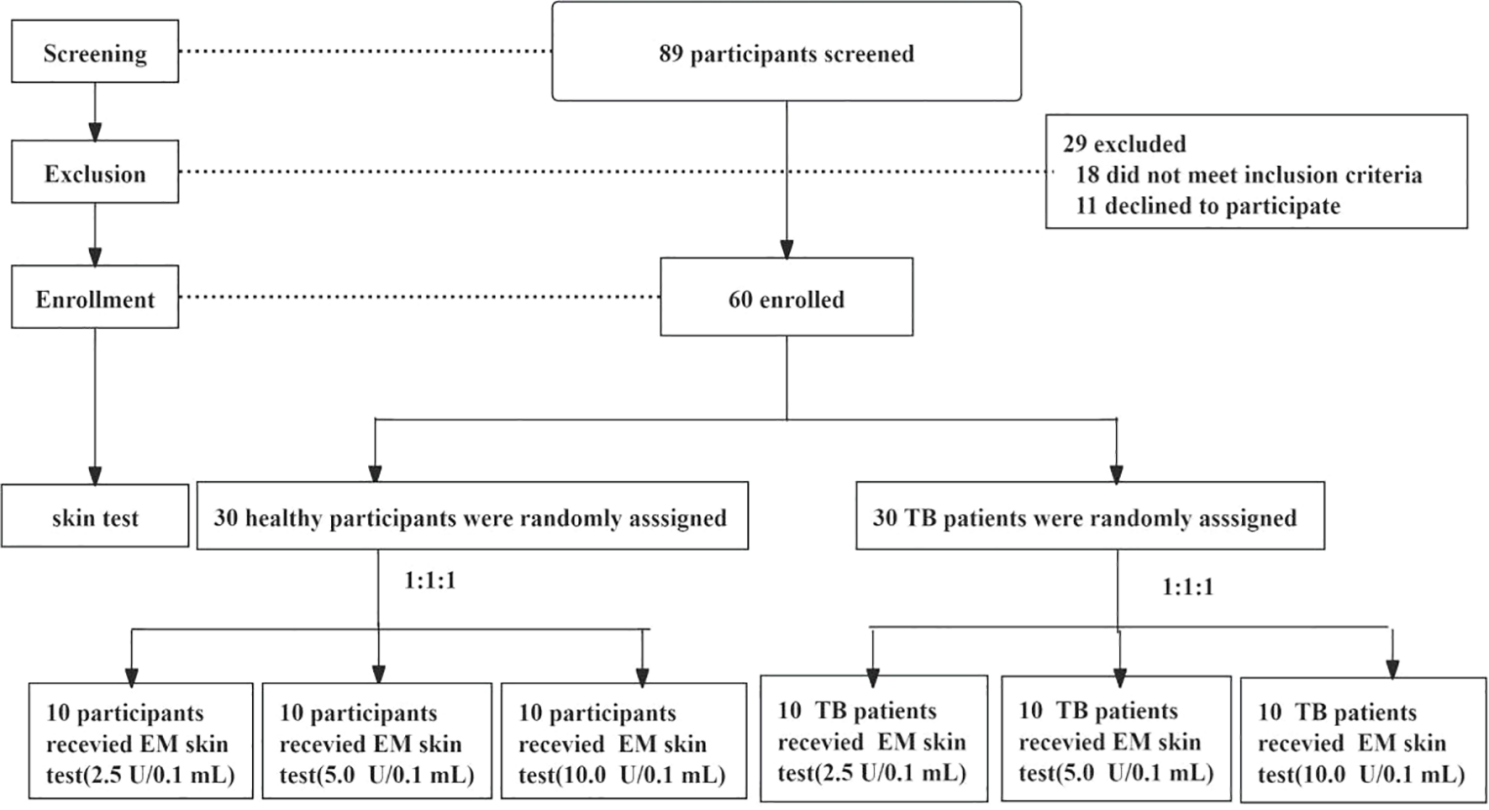
Trial profile. EM skin test= a recombinant ESAT6–MPT64 skin test for *Mycobacterium tuberculosis* infection.

A total of 89 individuals were screened for eligibility, of whom 60 participants were enrolled and constituted the Full Analysis Set (FAS). The cohort comprised 30 healthy controls (HC) and 30 patients with active pulmonary tuberculosis (ATB). All enrolled participants received the EM antigen injection. Fifty-nine participants (98.3%) completed the full study protocol. One participant (1.7%) in the medium-dose ATB cohort withdrew prematurely due to personal reasons unrelated to the investigational product or any adverse event (**Figure 1**).Baseline demographic and clinical characteristics were generally balanced across the three dose cohorts. The age distribution was comparable between groups, although a higher proportion of male participants was observed in the ATB group compared to HCs, reflecting the epidemiological characteristics of TB in the local population (**Table 1**).

**Table 1:** Demographic characteristics of the recruited tuberculosis patients, and healthy participants.

Demographic characteristic	General healthy participants			Tuberculosis patients		
	2.5 U/0.1 mL	5 U/0.1 mL	10 U/0.1 mL	2.5 U/0.1 mL	5 U/0.1 mL	10 U/0.1 mL
N	10	10	10	10	10	10
Sex						
Male	8 (80.0)	8 (80.0)	8 (80.0)	7 (70.0)	4 (40.0)	7 (70.0)
Female	2 (20.0)	2 (20.0)	2 (20.0)	3 (30.0)	6 (60.0)	3 (30.0)
Mean age,years (SD)	31.9 ± 8.5	29.2 ± 7.2	29.0 ± 5.2	44.7 ± 14.5	48.4 ± 16.0	35.8 ± 12.5
Mean height (SD),cm	165.4 ± 7.7	170.4 ± 4.6	169.9 ± 8.4	167.7 ± 6.5	164.2 ± 7.7	167.4 ± 5.1
Mean weight (SD),kg	62.66 ± 7.8	66.8 ± 9.3	69.0 ± 9.0	58.6 ± 12.5	55.0 ± 10.2	57.6 ± 9.1
BMI (kg/m^2^,Mean ± SD)	23.0 ± 3.3	22.9 ± 2.6	23.9 ± 2.7	20.7 ± 3.4	20.4 ± 3.5	20.5 ± 2.7
Ethnic group		10/0	10/0	10/0	10/0	10/0
Han	10 (100.0)	10 (100.0)	10 (100.0)	10 (100.0)	10 (100.0)	10 (100.0)
Other	0	0	0	0	0	0

SD, standard deviation.

### Safety and tolerability profile

The EM skin test demonstrated a favorable safety profile across all dose cohorts. No immediate hypersensitivity reactions were observed within the first 30 minutes post-injection. Importantly, no Grade 3 or 4 (severe or life-threatening) adverse events (AEs) were reported, and no participants discontinued the study due to AEs. Two serious adverse events (SAEs) occurred in the ATB cohort (incidence 6.7%); however, following independent adjudication, both were deemed unrelated to the investigational product.

#### Local reactions

Injection-site reactions were generally mild, dose-dependent, and manageable. The incidence of injection-site swelling was 10% (1/10), 50% (5/10), and 50% (5/10) in the low-, medium-, and high-dose groups, respectively. Injection-site pain was reported by 30% (3/10) of participants in the medium-dose group and 10% (1/10) in the high-dose group. No participants reported injection-site pruritus, ulceration, or necrosis.

#### Systemic adverse events

Systemic AEs were infrequent and self-limiting. In the low-dose group, two participants (20%) reported systemic AEs, specifically fatigue and nausea. In the medium-dose group, one participant (10%) reported a systemic AE. All reported AEs were Grade 1-2 in intensity and resolved spontaneously without medical intervention within 7 days. Overall, drug-related systemic adverse events were infrequent and primarily mild, indicating a low incidence of systemic reactogenicity (**Table 2**).

**Table 2:** Adverse Events and non-adverse events in recruited tuberculosis patients, and healthy participants in the full analysis set.

Event classification	General healthy participants			Tuberculosis patients		
	2.5 U/0.1 mL	5 U/0.1 mL	10 U/0.1 mL	2.5 U/0.1 mL	5 U/0.1 mL	10 U/0.1 mL
Adverse events (AEs)						
Pain	1 (10.0)	0 (0.0)	0 (0.0)	0 (0.0)	3 (30.0)	1 (10.0)
Swelling	1 (10.0)	0 (0.0)	0 (0.0)	1 (10.0)	5 (50.0)	5 (50.0)
Fever	0 (0.0)	0 (0.0)	0 (0.0)	0 (0.0)	0 (0.0)	1 (10.0)
Rash	0 (0.0)	0 (0.0)	0 (0.0)	1 (10.0)	0 (0.0)	0 (0.0)
Pruritus	0 (0.0)	0 (0.0)	0 (0.0)	0 (0.0)	0 (0.0)	0 (0.0)
Injury	0 (0.0)	0 (0.0)	0 (0.0)	0 (0.0)	0 (0.0)	2 (20.0)
Non-Adverse Events (Non-AEs)						
Fever	0 (0.0)	1 (10.0)	0 (0.0)	0 (0.0)	0 (0.0)	0 (0.0)
Fatigue	1 (10.0)	0 (0.0)	0 (0.0)	0 (0.0)	0 (0.0)	0 (0.0)
Nausea	1 (10.0)	0 (0.0)	0 (0.0)	0 (0.0)	0 (0.0)	0 (0.0)
Myalgia	0 (0.0)	0 (0.0)	0 (0.0)	0 (0.0)	0 (0.0)	1 (10.0)

#### Laboratory parameters

No clinically relevant abnormalities were observed in vital signs, physical examinations, or clinical laboratory parameters (hematology and serum biochemistry) throughout the study period (**Table 3**).

**Table 3:** Injection-site reactions and non-injection-site reactions in recruited tuberculosis patients, and healthy participants in the full analysis set.

Classification of adverse events	General healthy participants									Tuberculosis patients								
	2.5 U/0.1 mL		5 U/0.1 mL		10 U/0.1 mL		Total		P-value	2.5 U/0.1 mL		5 U/0.1 mL		10 U/0.1 mL		Total		P-value
	n(%)	Episodes	n(%)	Episodes	n(%)	Episodes	n(%)	Episodes		n(%)	Episodes	n(%)	Episodes	n(%)	Episodes	n(%)	Episodes	
All AEs	4(40.0)	8	3(30.0)	4	4(40.0)	9	11(36.7)	21	1.000	9(90.0)	18	7(70.0)	19	8(80.0)	21	24(80.0)	58	0.847
AEs within 30 min post-skin test	0(0.0)	0	0(0.0)	0	0(0.0)	0	0(0.0)	0	–	0(0.0)	0	0(0.0)	0	0(0.0)	0	0(0.0)	0	–
Injection site AEs	1(10.0)	2	0(0.0)	0	0(0.0)	0	1(3.3)	2	1.000	2(20.0)	2	5(50.0)	8	5(50.0)	9	12(40.0)	19	0.348
Non-injection site AEs	4(40.0)	6	3(30.0)	4	4(40.0)	9	11(36.7)	19	1.000	7(70.0)	16	6(60.0)	11	7(70.0)	12	20(66.7)	39	1.000
Grade ≥3 AEs	0(0.0)	0	0(0.0)	0	1(10.0)	1	1(3.3)	1	1.000	1(10.0)	1	2(20.0)	2	1(10.0)	1	4(13.3)	4	1.000
AEs leading to trial discontinuation	0(0.0)	0	0(0.0)	0	0(0.0)	0	0(0.0)	0	–	0(0.0)	0	0(0.0)	0	0(0.0)	0	0(0.0)	0	–
Treatment-Related AEs	3(30.0)	4	1(10.0)	1	0(0.0)	0	4(13.3)	5	0.286	2(20.0)	2	5(50.0)	8	5(50.0)	10	12(40.0)	20	0.348
AEs within 30 min post-skin test	0(0.0)	0	0(0.0)	0	0(0.0)	0	0(0.0)	0	–	0(0.0)	0	0(0.0)	0	0(0.0)	0	0(0.0)	0	–
Injection site AEs	1(10.0)	2	0(0.0)	0	0(0.0)	0	1(3.3)	2	1.000	2(20.0)	2	5(50.0)	8	5(50.0)	9	12(40.0)	19	0.348
Non-injection site AEs	2(20.0)	2	1(10.0)	1	0(0.0)	0	3(10.0)	3	0.754	0(0.0)	0	0(0.0)	0	1(10.0)	1	1(3.3)	1	1.000
Grade ≥3 AEs	0(0.0)	0	0(0.0)	0	0(0.0)	0	0(0.0)	0	–	0(0.0)	0	1(10.0)	1	0(0.0)	0	1(3.3)	1	1.000
AEs leading to trial discontinuation	0(0.0)	0	0(0.0)	0	0(0.0)	0	0(0.0)	0	–	0(0.0)	0	0(0.0)	0	0(0.0)	0	0(0.0)	0	–
AEs within 30 min post-skin test	3(30.0)	4	2(20.0)	3	4(40.0)	9	9(30.0)	16	0.879	7(70.0)	16	6(60.0)	11	6(60.0)	11	19(63.3)	38	1.000

### Kinetics of cutaneous immune responses

**Figure 2:**
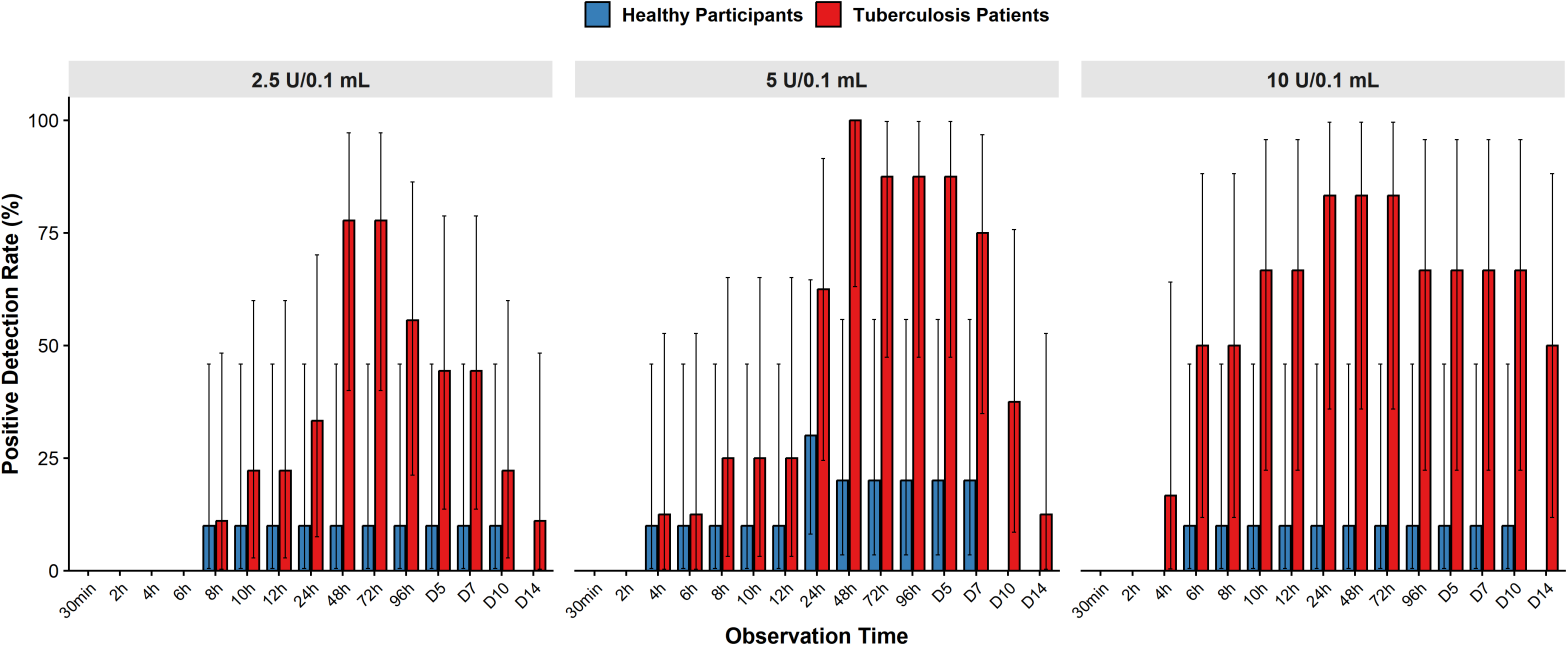
Negative concordance rate of the recruited tuberculosis patients, and healthy participant.

In the active TB cohort, the EM antigen elicited a rapid recall response. Palpable induration or erythema was observed as early as 4 to 8 hours post-injection. Specifically, positive reactions were first detected at 8 hours in the low-dose group, and at 4 hours in both the medium- and high-dose groups. The positive detection rate (sensitivity) varied over time. Across all dose groups, peak sensitivity was consistently observed at the 48-hour time point, with detection rates of 77.8%, 100.0%, and 83.3% in the low-, medium-, and high-dose cohorts, respectively (**Figure 2**). By 72 hours, the corresponding rates modulated to 77.8%, 87.5%, and 83.3%, respectively (**Supplementary Table S1**).The aggregate positive detection rate among all ATB patients peaked at 87.0% at 48 hours and showed a slight decline to 82.6% at 72 hours. Inter-group statistical analysis revealed significant heterogeneity in detection rates only at the 6-hour time point (*P* = 0.039), where the low-dose group exhibited a significantly delayed response compared to the high-dose group.

### Diagnostic performance (ROC analysis)

**Figure 3:**
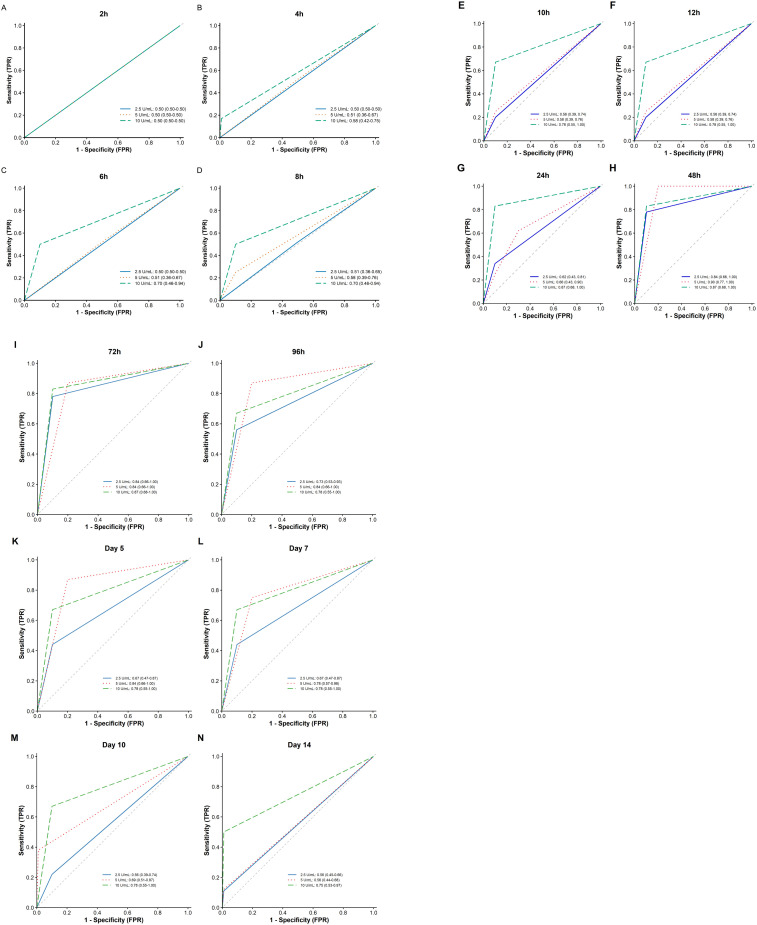
**(A–N)** Estimated AUC for diagnosis with the EM skin test with different indicators at different doses and time points. AUC, area under the curve; EM, ESAT6–MPT64.

Receiver Operating Characteristic (ROC) curve analysis demonstrated that the diagnostic performance of the EM skin test was optimal between 48 and 72 hours post-injection. The Area Under the Curve (AUC) values consistently exceeded 0.80 across all dose groups, indicating robust diagnostic accuracy (**Figure 3**). The AUC estimates were accompanied by 95% confidence intervals to reflect the precision of the diagnostic performance estimates. Specifically, at the critical 48-hour time point: The low-dose cohort achieved an AUC of 0.84.The medium-dose cohort reached a peak AUC of 0.90.The high-dose cohort maintained an AUC of 0.87 (a value sustained from 24 to 72 hours) (**Table 2**). These findings indicate that the EM skin test demonstrates good discriminatory ability for distinguishing ATB patients from healthy controls.

### Concordance with interferon-γ release assays

Agreement between the EM skin test and the reference IGRA (T-SPOT.TB) was evaluated based on the optimal 48-hour readout. In Healthy Controls (Specificity): The baseline IGRA negativity rate was 90.0% (9/10) across all dose cohorts. In the low-dose cohort, EM test results demonstrated perfect agreement with IGRA at all timepoints (κ= 1.0). In the medium-dose cohort, complete concordance (κ = 1.0) was observed from 4 to 12 hours, with moderate agreement (0.4 < κ < 0.75) persisting between 24 hours and Day 7. In the high-dose cohort, complete concordance (κ = 1.0) was observed from 24 hours to Day 10.In ATB Patients (Sensitivity): The baseline IGRA positivity rates were 77.8% (7/9), 100.0% (8/8), and 66.7% (4/6) for the low-, medium-, and high-dose groups, respectively. Low-dose cohort: EM skin test positivity at 48 and 72 hours showed 100% concordance with IGRA results. Medium-dose cohort: Both assays achieved 100% positivity at 48 hours. From 72 hours through Day 5, both assays maintained a positivity rate of 87.5%, indicating sustained high concordance. High-dose cohort: Poor agreement (κ < 0.40) was observed at several early and late time points. This divergence suggests a potential kinetic mismatch or saturation effect in the high-dose group compared to the systemic T-cell response measured by IGRA.

## Discussion

In this Phase 1 randomized clinical trial, we provide the first-in-human evaluation of the novel recombinant ESAT6-MPT64 (EM) skin test. Our findings demonstrate that this novel fusion protein possesses a favorable safety profile and elicits robust antigen-specific cell-mediated immune responses in patients with active tuberculosis. While the primary objective was to establish safety, the preliminary efficacy data-characterized by an AUC exceeding 0.80 and sensitivity greater than 87%-suggests that the EM skin test holds significant promise as a scalable, specific alternative to traditional PPD-based diagnostics and a viable competitor to existing IGRA platforms.

Our data indicate that the EM skin test achieves high diagnostic accuracy, effectively ruling out infection in healthy controls (negative concordance >83%) while maintaining high sensitivity in active TB patients. This performance aligns closely with established benchmarks for other next-generation skin tests, such as the C-TST and Diaskintest (17–19), which rely solely on the ESAT6-CFP10 (EC) antigen combination (20). However, our study distinguishes itself by validating the inclusion of MPT64. While ESAT-6 and CFP-10 are potent immunogens, relying on a limited epitope repertoire may theoretically compromise sensitivity in populations with diverse HLA backgrounds. MPT64, encoded by the RD2 region, is a highly specific, secreted antigen capable of inducing strong DTH responses (11). The comparable, and in some metrics superior, performance observed in our cohort suggests that the EM fusion protein successfully preserves the immunogenicity of ESAT-6 while potentially broadening the antigenic coverage through MPT64, offering a “safety net” for detecting infections that might be missed by RD1-restricted assays.

Defining the optimal reading window is critical for clinical utility. We observed that the highest diagnostic concordance and peak signal-to-noise ratios (erythema and induration) occurred between 48 and 72 hours post-injection. This timeframe yields robust discriminatory power (AUC approaching 0.90) and aligns perfectly with the standard operational workflows for TST. Notably, regarding dose-response relationships, significant heterogeneity in detection rates was observed only at the immediate post-injection phase (6 hours). At all subsequent clinically relevant time points (24-72 hours), diagnostic performance was stable across the low-, medium-, and high-dose cohorts. These observations suggest that the EM antigen demonstrates a relatively stable diagnostic performance across the evaluated dose range, which may facilitate flexibility in dose selection in future clinical development.

A key advantage of recombinant skin tests is their potential to match the specificity of IGRAs by avoiding BCG cross-reactivity. In our study, the EM skin test demonstrated substantial concordance (κ > 0.60) with T-SPOT.TB in active TB patients, reinforcing that the skin reaction reflects a genuine, specific T-cell recall response. In healthy controls, while moderate agreement was observed in some sub-cohorts, this likely reflects the small sample size inherent to a Phase 1 design rather than a biological defect. Crucially, the high negative concordance rates suggest that the EM antigen is not confounded by prior BCG vaccination, a pervasive issue with PPD that limits the utility of the TST in China and other high-burden nations (21).

The safety evaluation confirms that the EM skin test is well tolerated. The overall incidence of adverse events (AEs) was 13.3% in healthy volunteers and 40.0% in TB patients. The higher incidence of AEs in the TB cohort likely reflects heightened antigen-specific immune responsiveness rather than intrinsic toxicity of the investigational antigen. It is critical to interpret the higher AE rate in TB patients as a reflection of heightened specific immune memory (a desired mechanism of action) rather than intrinsic toxicity. All reported AEs were mild (Grade 1-2), transient, and predominantly localized (swelling/pain), resolving spontaneously without sequelae. This safety profile compares favorably with existing products. For instance, the Diaskintest has been associated with necrotic reactions in up to 14% of cases (22, 23), and the C-TST has reported injection-site reaction rates of approximately 27.8% (24). In our trial, no necrosis, lymphangitis, or severe systemic reactions (Grade 3/4) were observed. The absence of a clear dose-response relationship in AE frequency further supports a wide safety margin for the EM antigen, facilitating its transition to larger-scale trials.

Our study has limitations inherent to its Phase 1 design. The primary constraint is the small sample size, which, while sufficient for safety assessment, limits the statistical power to definitively validate diagnostic sensitivity across diverse subpopulations (e.g., pediatric, geriatric, or immunocompromised patients). Therefore, in this Phase 1 setting, the reported AUC and sensitivity values should be interpreted as preliminary indicators of diagnostic potential rather than definitive measures of diagnostic accuracy. Additionally, the open-label nature of the study may introduce observer bias in subjective reading, although the use of objective measurement tools (calipers) mitigates this risk. Finally, as this study focused on active TB and healthy controls, future Phase 2/3 trials are necessary to evaluate the test’s performance in Latent TB Infection (LTBI) cohorts, which represent the primary target for preventive screening (25).

The development of the EM skin test holds substantial implications for global tuberculosis control. By combining the operational simplicity and low cost of a skin test with the high specificity of an IGRA, EM addresses the critical need for scalable screening tools in resource-limited, BCG-vaccinated populations (26–28). Furthermore, the use of recombinant technology ensures consistency and scalability, overcoming the supply chain and batch variability issues associated with PPD.

In conclusion, this trial demonstrates that the recombinant EM skin test possesses an excellent safety profile and robust diagnostic potential. The inclusion of MPT64 alongside ESAT-6 offers a novel antigenic formulation that performs comparably to IGRAs. These findings justify the progression to Phase 2 and Phase 3 clinical evaluations to establish its real-world effectiveness as a frontline tool for TB elimination.

## Data Availability

The original contributions presented in the study are included in the article/**Supplementary Material**. Further inquiries can be directed to the corresponding author.
