# Correction: Safety, tolerability, and concordance with interferon-γ release assays of a recombinant ESAT6–MPT64 skin test: a phase 1 randomized clinical trial

**DOI:** 10.3389/fimmu.2026.1863033

**Published:** 2026-04-29

**Authors:** Xichao Ou, Guan Liu, Baozhen Peng, Yuehua Li, Hegui Yan, Naihui Chu, Bing Zhao, Yanlin Zhao, Juan Du

**Affiliations:** 1 National Key Laboratory of Intelligent Tracking and Forecasting for Infectious Diseases, National Center for Tuberculosis Control and Prevention, Chinese Center for Disease Control and Prevention (Chinese Academy of Preventive Medicine), Beijing, China; 2 Phase I Clincal Trial Center, Wuhan Pulmonary Hospital(Wuhan Institute for tuberculosis control), Wuhan, China; 3 Tuberculosis Department, Beijing Chest Hospital, Capital Medical University, Beijing, China

**Keywords:** diagnostic accuracy, ESAT6-MPT64, IGRA concordance, MPT64, mycobacterium tuberculosis, phase 1 clinical trial, recombinant skin test

Author “Juan Du” was erroneously assigned as equal contributing author. Author “Guan Liu” was erroneously omitted as equal contributing author.

The original version of this article has been updated.

