# Supplementary material for: Safety, tolerability, and concordance with interferon-γ release assays of a recombinant ESAT6–MPT64 skin test: a phase 1 randomized clinical trial
Source: Front Immunol. 2026 Apr 21;17:1780294. doi: 10.3389/fimmu.2026.1780294 (PMC13169258; doi:10.3389/fimmu.2026.1780294)
Supplement: Supplementary file 1 [file Table1.docx]

**Table S1. Negative Concordance Rate of the Recruited Tuberculosis Patients, and Healthy Participants**

| **Skin Test Observation Time** | **General Healthy participants** | | | | | | **Tuberculosis Patients** | | | | | |
| --- | --- | --- | --- | --- | --- | --- | --- | --- | --- | --- | --- | --- |
|  | **EM Skin Test,2.5 U/0.1 mL** | | **EM Skin Test,5 U/0.1 mL** | | **EM Skin Test,10 U/0.1 mL** | | **EM Skin Test,2.5 U/0.1 mL** | | **EM Skin Test,5 U/0.1 mL** | | **EM Skin Test,10 U/0.1 mL** | |
|  | **Positive Detection N（%）** | **95%CI** | **Positive Detection N（%）** | **95%CI** | **Positive Detection N（%）** | **95%CI** | **Positive Detection N（%）** | **95%CI** | **Positive Detection N（%）** | **95%CI** | **Positive Detection N（%）** | **95%CI** |
| 30 minutes | 0(0.0) | - | 0(0.0) | - | 0(0.0) | - | 0(0.0) | - | 0(0.0) | - | 0(0.0) | - |
| 2h | 0(0.0) | - | 0(0.0) | - | 0(0.0) | - | 0(0.0) | - | 0(0.0) | - | 0(0.0) | - |
| 4h | 0(0.0) | - | 1(10.0) | 0.5-45.9 | 0(0.0) | - | 0(0.0) | - | 1(12.5) | 0.3-52.7 | 1(16.7) | 0.4-64.1 |
| 6h | 0(0.0) | - | 1(10.0) | 0.5-45.9 | 1(10.0) | 0.50-45.9 | 0(0.0) | - | 1(12.5) | 0.3-52.7 | 3(50.0) | 11.8-88.2 |
| 8h | 1(10.0) | 0.50- 45.88 | 1(10.0) | 0.5-45.9 | 1(10.0) | 0.5-45.9 | 1(11.1) | 0.3-48.3 | 2(25.0) | 3.2-65.1 | 3(50.0) | 11.8-88.2 |
| 10h | 1(10.0) | 0.50-45.88 | 1(10.0) | 0.5-45.9 | 1(10.0) | 0.5-45.9 | 2(22.2) | 2.8-60.0 | 2(25.0) | 3.2-65.1 | 4(66.7) | 22.3-95.7 |
| 12h | 1(10.0) | 0.50- 45.88 | 1(10.0) | 0.5-45.9 | 1(10.0) | 0.5-45.9 | 2(22.2) | 2.8-60.0 | 2(25.0) | 3.2-65.1 | 4(66.7) | 22.3-95.7 |
| 24h | 1(10.0) | 0.50-45.88 | 3(30.0) | 8.1-64.6 | 1(10.0) | 0.5-45.9 | 3(33.3) | 7.5-70.1 | 5(62.5) | 24.5-91.5 | 5(83.3) | 35.9-99.6 |
| 48h | 1(10.0) | 0.50-45.88 | 2(20.0) | 3.5-55.8 | 1(10.0) | 0.5-45.9 | 7(77.8) | 40.0-97.2 | 8(100.0) | 63.1-100.0 | 5(83.3) | 35.9-99.6 |
| 72h | 1(10.0) | 0.50- 45.88 | 2(20.0) | 3.5-55.8 | 1(10.0) | 0.5-45.9 | 7(77.8) | 40.0-97.2 | 7(87.5) | 47.4-99.7 | 5(83.3) | 35.9-99.6 |
| 96h | 1(10.0) | 0.50-45.88 | 2(20.0) | 3.5-55.8 | 1(10.0) | 0.5-45.9 | 5(55.6) | 21.2-86.3 | 7(87.5) | 47.4-99.7 | 4(66.7) | 22.3-95.7 |
| D5 | 1(10.0) | 0.50-45.88 | 2(20.0) | 3.5-55.8 | 1(10.0) | 0.5-45.9 | 4(44.4) | 13.7-78.8 | 7(87.5) | 47.4-99.7 | 4(66.7) | 22.3-95.7 |
| D7 | 1(10.0) | 0.50- 45.88 | 2(20.0) | 3.5-55.8 | 1(10.0) | 0.5-45.9 | 4(44.4) | 13.7-78.8 | 6(75.0) | 34.9-96.8 | 4(66.7) | 22.3-95.7 |
| D10 | 1(10.0) | 0.50-45.88 | 0(0.0) | - | 1(10.0) | 0.5-45.9 | 2(22.2) | 2.8-60.0 | 3(37.5) | 8.52-75.71 | 4(66.7) | 22.3-95.7 |
| D14 | 0(0.0) | - | 0(0.0) | - | 0(0.0) | - | 1(11.1) | 0.3-48.3 | 1(12.5) | 0.3-52.7 | 3(50.0) | 11.8-88.2 |

Note For the positive detection rate: the number outside the parentheses represents the count of occurrences; the percentage in the parentheses is calculated by taking the number of enrolled subjects in each group of healthy people in PPS as the denominatora and the number outside the parentheses as the numerator.
